# Phonon Confinement Induced Non-Concomitant Near-Infrared Emission along a Single ZnO Nanowire: Spatial Evolution Study of Phononic and Photonic Properties

**DOI:** 10.3390/nano7110353

**Published:** 2017-10-28

**Authors:** Po-Hsun Shih, Tai-Yue Li, Yu-Chen Yeh, Sheng Yun Wu

**Affiliations:** Department of Physics, National Dong Hwa University, Hualien 97401, Taiwan; libra.kevin.t@gmail.com (P.-H.S.); tim312508@gmail.com (T.-Y.L.); 410314213@gms.ndhu.edu.tw (Y.-C.Y.)

**Keywords:** ZnO nanowire, confocal Raman spectroscopy, EDS mapping, NIR luminescence

## Abstract

The impact of mixed defects on ZnO phononic and photonic properties at the nanoscale is only now being investigated. Here we report an effective strategy to study the distribution of defects along the growth direction of a single ZnO nanowire (NW), performed qualitatively as well as quantitatively using energy dispersive spectroscopy (EDS), confocal Raman-, and photoluminescence (PL)-mapping technique. A non-concomitant near-infrared (NIR) emission of 1.53 ± 0.01 eV was observed near the bottom region of 2.05 ± 0.05 μm along a single ZnO NW and could be successfully explained by the radiative recombination of shallowly trapped electrons $V_{O}^{**}$ with deeply trapped holes at $V_{Zn}^{\prime \prime }$. A linear chain model modified from a phonon confinement model was used to describe the growth of short-range correlations between the mean distance of defects and its evolution with spatial position along the axial growth direction by fitting the E_2_^H^ mode. Our results are expected to provide new insights into improving the study of the photonic and photonic properties of a single nanowire.

## 1. Introduction

Zinc oxide (ZnO) nanowires (NWs) are of great interest for the development of devices in various applied fields that include a direct and wideband gap, optoelectronic devices like light-emitting diodes, strong ultraviolet emissions, and solid-state lasers [1,2,3,4,5]. Recent studies on ZnO NWs have slightly shifted the focus to different aspects that include the Zn vacancy induced green luminescence [6], oxygen induced strain effect [7], and the influence of short-range correlation in phonon confinement [8]. Despite the practical importance, the current knowledge of the phonon vibration properties of ZnO nanocrystals is rather limited. Confocal Raman spectroscopy is a nondestructive measurement of the phonon vibration properties of ZnO nanocrystals [9,10]. All described polar- and nonpolar- optical phonon modes of ZnO nanocrystals have been reported and revealed frequency shifts compared to their positions in the spectrum from bulk ZnO [11,12]. The origin of these shifts of the phonon vibration mode in ZnO nanocrystals is still under debate. The most controversial issue in nano scale ZnO is the determination of the chemical and crystallographic origin of the visible bands due to deep band emission. This emission can be explained using two main contributions, near-band-edge emission (UV range) and defect-related emissions (visible). For green luminescence (GL), some groups attributed this phenomenon to the oxygen vacancies and assigned emission centered at about 490 to 530 nm [13,14,15,16]. Furthermore, the localized emission at the surface interprets this green band as even more complicated [17,18,19]. Recently, the observation of concomitant near-infrared (NIR) photoluminescence from ZnO has attracted a lot of attention. Lauer et al. [20] reported a 1.70 eV NIR emission, observed at higher temperature oxygen-annealed ZnO, and a broad emission band covering the NIR, and the visible spectrum was found to center at 1.55 to 1.9 eV for various gas- or thermal-annealed ZnO [21,22,23]. The above studies indicated that the NIR emission was normally a concomitant of the broad visible emission. How to investigate the origin of NIR emission is still a challenge due to the multi-contributions of shapes, size distributions, thermal effects, surface effects, agglomeration, and strain, which have resulted in a complicated state, in which the experimental pieces of information could be merged and can not be compared with one another. An in situ confocal Raman mapping measurement along a single ZnO NW, therefore, becomes important and provides a reasonable possibility for probing the size effect and confinement effect. In our previous report [24,25], we proposed that metal-oxide NWs grown below the melting temperatures are attributed to the short-circuit diffusion mechanism. How to observe and analyze the atomic diffusion and phonon confinement in the metal-oxide nanowire, therefore, becomes important along the growth direction of a single NW, using optical measurements. A Raman mapping technique was employed to investigate the phonon and geometric properties of a single ZnO NW [7,8].

In most of these various synthesis methods, various catalysts or auxiliaries were utilized in these fabrication processes. Residual contaminations could be found on the surface or at the edge of the nanomaterials, with the result that it is difficult to observe the essential properties of ZnO. Therefore, the synthesis of nanostructures without a catalytic template or using the self-catalytic behavior of the material would be of interest. To solve these problems, a new one-step growth method is developed using the chemical vapor deposition (CVD) process. The availability of large quantities of well-aligned in-plane ZnO NWs in a single crystalline form is extremely important for the development of high-efficiency, short wavelength optoelectronic nano-devices. In this study, a single ZnO NW was synthesized using a Ti-assisted CVD method to avoid the appearance of catalytic contamination. A confocal Raman spectrometer has been used to investigate the phonon confinement effect of phonon-vibration- and NIR luminescence-mapping on the evolution of defects along the growth direction of a single ZnO NW, without any post-annealing and non-element doped processes.

## 2. Results

### 2.1. Sample Characterization and TEM Analysis

**Figure 1:**
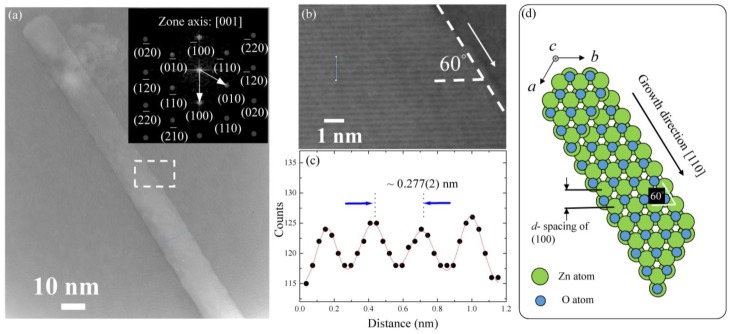
(**a**) Transmission electron microscopy (TEM) images of an individual straight ZnO nano-wire (NW). The inset to (**a**) is the electron diffraction pattern of a selected area of the nanowire, revealing the hexagonal structure of ZnO; (**b**) high-resolution transmission electron microscopy (HR-TEM) image of the selection region (marked in (**a**)) of ZnO NW; (**c**) height-position intensity along the line taken from HR-TEM (marked in (**b**)); and (**d**) schematic plot of the atomic model for a single ZnO NW.

Transmission electron microscopy (TEM) further investigated the morphologies of a single ZnO NW. Figure 1a shows a TEM image of an individual straight ZnO NW. The image reveals that most of the wire was straight, and the diameter along the growth direction is relatively uniform, with a mean value of 13 ± 1 nm. The corresponding selected-area electron diffraction (SAED) pattern, shown in the inset of Figure 1a, taken from a single nanowire, reveals the single-crystalline nature of the sample. The Bragg diffraction spots correspond to the [001] reflection of the wurtzite crystal structure of ZnO NW. The pattern of the main spots can easily be indexed based on a space group of *P6_3_mc*. A high-magnification enlargement of a selected area of the high-resolution TEM image is shown in Figure 1b. It can be seen that the normal direction of the planes is not parallel to the growth direction of the ZnO NW, with a canted angle of 60 degrees. A gray-level analysis was used to extract the scattering intensity, which can be fit using a multi-Gaussian function to obtain the average plane spacing of (100). Figure 1c shows the fitting result, in which the solid circle and the solid blue line show the experimental data and fitting curve, respectively. The fitted distance is near ~0.277 ± 0.002 nm, corresponding to the *d*-spacing of (100) planes of the ZnO hexagonal structure. The lattice parameter of the hexagonal structure can be calculated using the relationship $\;\frac{1}{d_{hkl}^{2}}=\frac{4}{3}\left(\frac{h^{2}+hk+k^{2}}{a^{2}}\right)+\frac{l^{2}}{c^{2}}$, where *d_hkl_* is a spacing between two planes of (*hkl*), *a* and *c* are lattice parameters, and *h*, *k,* and *l* are the Miller indices. The obtained lattice parameter *a* is near ~0.32 ± 0.02 nm. Since the *c*-axis of the hexagonal structure is perpendicular to the scattering plane, we could not measure the lattice constant *c.* Based on this analysis, a schematic plot of the atomic model of a single ZnO NW is shown in Figure 1d. The growth mechanism of the ZnO NWs is discussed in the previous report based on the above structural analysis, showing a short-circuit diffusion mechanism along the growth direction [26]. The crystal uniformity along the growth direction depending on the fabrication conditions, substrate, native defects (such as oxygen vacancies, interstitials, and antisite) or extrinsic defects can be introduced, thus leading to the control of the phonon confinement and the luminescence response in the visible regime of the ZnO nanocrystals [7].

### 2.2. High Spatial Resolution of Energy Dispersive Spectroscopy Mapping

**Figure 2:**
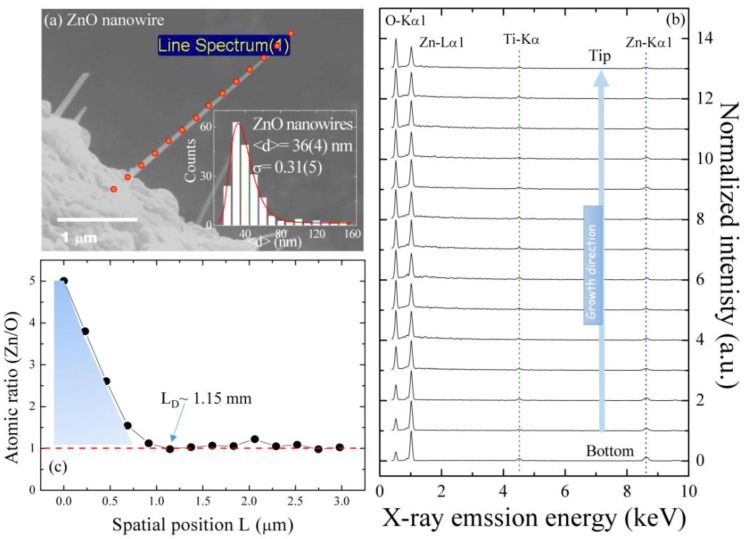
(**a**) Field-emission scanning electron microscopy (FE-SEM) image of a single ZnO NW; (**b**) corresponding energy dispersive spectroscopy (EDS) spectra along the growth direction; and (**c**) the atomic ratio Zn/O versus the position. The inset to (**a**) shows the diameter distribution taken from a portion of the SEM image.

Taking advantage of the high spatial resolution of the energy dispersive spectroscopic mapping technique, the evolution of the zinc and oxygen along a single ZnO NW is analyzed. Figure 2a displays a field-emission scanning electron microscopy (FE-SEM) image of a single ZnO NW, and the energy dispersive spectroscopy (EDS) mapping was taken along the growth direction, where the red circles indicate the spatial positions that correspond to the spot-mode EDS spectrum shown in Figure 2b. The solid red line in the inset of Figure 2a describes the asymmetric distribution of the diameter fitted with a lognormal distribution function: $f\left(d\right)=\frac{1}{\sqrt{2\pi }d\sigma }exp\left[-\frac{{\left(lnd-ln<d>\right)}^{2}}{2\sigma^{2}}\right]$, where <*d*> is the mean diameter and *σ* is the standard deviation of the function. The fitted values of the diameters and the standard deviation are 36 ± 4 nm and 0.31 ± 0.05 nm for ZnO NWs, respectively. The observed wide distribution and agglomeration behavior of the nanowires can significantly affect the phonon properties. The corresponding EDS results point out that the elemental spectra at various spatial positions are associated with a series of elemental zinc and oxygen constituents, which can be assigned to O-Kα_1_, Zn-Lα_1_, Ti-Kα, and Zn-Kα_1_, respectively. The estimated atomic percent ratio of zinc/oxygen (obtained using EDS) with respect to various spatial positions is plotted in Figure 2c, revealing a decreasing of the ratio, moving from the bottom *L* = 0 to the position of *L*_D_ = 1.15 µm, and showing a monotonic trend of the ratio toward the nanowire tip. The decrease in the ratio of Zn/O (larger than 1) in the range of length *L* = 0 to 1.15 µm reveals the existence of oxygen vacancies, named the intermediated ZnO_1-*x*_ boundary region between the Zn small grains and the single crystalline ZnO NW. Indeed, it is highly probable that during short-circuit diffusion growth, the nanowire becomes poor in oxygen, favoring the formation of oxygen moving toward the nanowire tip. Although the EDS data do not convincingly reveal oxygen deficiencies, several groups have reported the formation of oxygen deficiencies within ZnO nanocrystals under high annealing temperatures, even in the ambient atmosphere due to the lower formation energy of an oxygen vacancy in ZnO.

### 2.3. Spatial Scanning Confocal Raman Measurements along a Single ZnO NW

**Figure 3:**
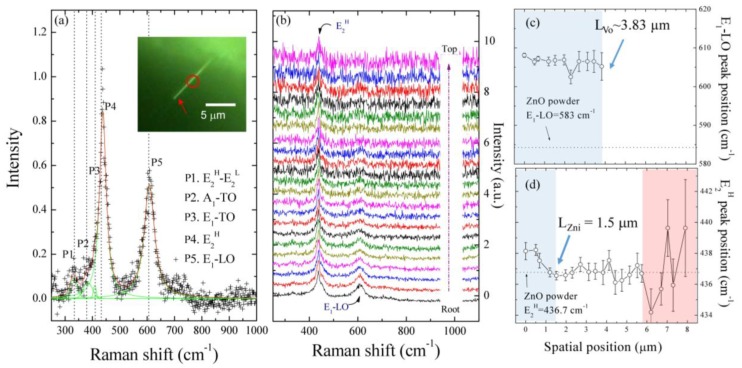
Raman spectra taken (**a**) from the bottom and (**b**) along the growth direction of a single ZnO NW. The inset to (**a**) shows the corresponding optical image of the ZnO NW; (**c**,**d**) peak position of the E_1_-LO and E_2_^H^ modes along the growth direction of the ZnO NW, respectively.

To gain insight on the microscopic origin of the defect inducing the shifting of some related phonon modes, we propose a confined confocal Raman and photoluminescence study of a single ZnO NW. Figure 3a shows a Raman spectrum taken at the root (red arrow in the inset) of the ZnO NW. The inset shows the corresponding optical image of the straight ZnO NW, with a length of 7.90 μm. A multi-Voigt function can describe the peak profiles. The fitting results are shown in Table 1. It can be seen that there are two strong peaks at 438.1 and 608.1 cm^−1^, corresponding to the E_2_^H^ mode and E_1_-LO mode, and three weak peaks at 331.9, 383.4, and 408.7 cm^−1^, corresponding with the A_1_ (acoustic overtone), A_1_-TO, and E_1_-TO modes, respectively [27]. The A_1_ and E_1_ modes represent the vibration of atoms parallel and perpendicular to the hexagonal *c*-axis, respectively, and they are each further divided into different frequencies for the transverse optical (TO) and longitudinal optical (LO) phonons due to long-range electrostatic forces. The low and high frequencies of the E_2_ mode (E_2_^L^ (low), E_2_^H^ (high)) are exclusively Raman-active and correspond to the vibration of the Zn and O sub-lattice, respectively. Compared with the Raman result of ZnO powder, as shown in the supporting information of Figure S1 and Table S1, the A_1_ (acoustic overtone), A_1_-TO, E_2_^H^, and E_1_-LO modes showed 2.0, 3.2, 1.4, and 24.8 cm^−1^ blue shifts, respectively, while the E_1_-TO mode showed a 3.2 cm^−1^ red shift. There are some possible explanations for these shifts. One is the finite size effect [28] that leads to a large band gap. Another one is that the nanostructures possess a piezoelectric effect, as the anisotropic confinement results in a different shift in each mode [29]. A possible one is that zinc interstitials and oxygen deficiencies cause a lattice distortion [29,30]. This assumption is in agreement with the EDS result that a mixed state of ZnO_1−*x*_ was proposed in the bottom region of a single ZnO NW, as published in our previous work [8]. Figure 3b shows a series of Raman spectra taken along the growth direction of a single ZnO NW. The shift and broadening of the scattering peaks of the E_1_-LO and E_2_^H^ modes were analyzed and summarized in Table 1, showing the possible related effects in different regions of a single ZnO NW. Raman spectra are also sensitive to structural defects in the synthesized ZnO NWs, and the presence of the E_1_-LO peak at around 608.1 cm^−1^ is attributed to the formation of defects such as oxygen vacancies and free carriers [29,30]. The intensity of the E_1_-LO mode rapidly weakened and then vanished at the spatial position *L* ~ 3.83 µm, namely, as a detectable length L_Vo_ of the oxygen vacancy state along a single ZnO NW, as shown in Figure 3c, whereas the intensity of E_2_^H^ mode remained unchanged. Figure 3d shows the spatial positions of the E_2_^H^ mode along the axial direction of the ZnO NW. At the bottom region of spatial positions, below L_Zni_ < 1.5 µm of the ZnO NW, a blue shift was observed, as compared with the ZnO powder of E_2_^H^ ~ 436.7 cm^−1^, and decreased with the increase of spatial scanning position. This anomalous phenomenon can be attributed to the defective state of Zn-interstitials [30,31,32,33,34]. As the scanned spatial position *L* increases from 1.5 to 5.7 µm, the peak shift and the related width becomes narrow, as tabulated in Table 1, due to the reduction in defect concentration. In the middle region, the small fluctuation in peak position and width can be attributed to a uniform diameter and a stable defect concentration [35]. In the top region, higher than a scan position of 5.7 µm, the existence of poor crystalline was assumed to lead to the red shift due to the phonon confinement effect [36,37]. The blue shift and increased width can be attributed to the size effect and surface strain [37,38]. The ratio of the atomic number of surface atoms to inner atoms at the top of the ZnO NW is higher than that at the bottom, so the surface strain becomes important. Combing the Raman mapping results from the E_1_-LO and E_2_^H^ modes with spatial position *L* below 1.5 µm, a mixed state of Zn-interstitials and/or oxygen vacancies was observed that is consistent with the observation of EDS results. In the middle region of *L* = 1.5 to 3.83 µm, a logical comparison of the relative defect densities of Zn-interstitials and oxygen vacancies suggested that the former are less efficient than the latter because of the differences in their intrinsic nature and the physical accessibility of their defects.

### 2.4. NIR Emission Mapping along a ZnO NW

**Figure 4:**
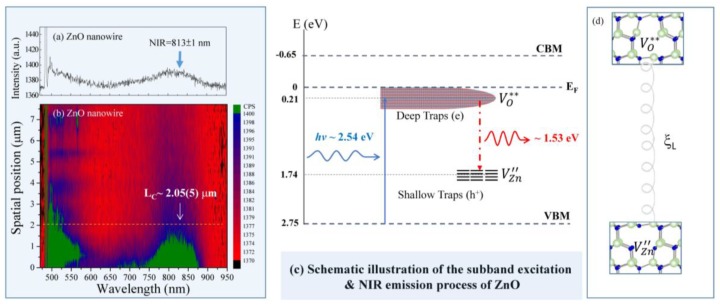
(**a**) Spatial position dependency of room temperature photoluminescence (PL) spectra, revealing a broader near infra-red (NIR) sub-band gap emission; (**b**) 2D plot of PL spectra along a single ZnO NW; (**c**) A schematic plot of the energy levels involved in the NIR luminescence using Kröger-Vink notation; and (**d**) A schematic plot of the local atomic geometry of $V_{O}^{**}$ and $V_{Zn}^{\prime \prime }$, respectively, where the mean distance between defects is defined as the correlation length $\xi_{L}$.

A spatial scanning confocal photoluminescence technique was used to investigate the evolution of the NIR emission along a single ZnO NW. Confocal photoluminescence microscopy is a helpful technique to investigate the optical properties of a single semiconducting nanowire. A photoluminescence spatial resolution of ~λ/2 is obtained by translating the collection aperture of the confocal microscopy arrangement, providing a method for obtaining spatial resolution in photoluminescence from semiconductor structures that are limited only by their optics rather than by carrier transport effects. It informs us about the energy states of impurities and defects, even at very low densities, which is helpful in understanding structural defects, crystallinity, oxygen vacancies, and zinc interstitials. Defects like oxygen vacancies at the grain boundaries and the presence of doping atoms in the surface and inter-granular layers can affect some physical properties (such as the optical properties) of nano-grained metal oxide materials. A selected single ZnO NW was excited by a 488 nm wavelength laser at room temperature and scanned along the growth direction from bottom to tip. A prominent peak, with the main peak at 813 ± 1 nm (~1.53 eV), exhibiting strong near-infrared emission was observed in the PL mapping spectra taken at *L* = 0, as shown in Figure 4a. A 2D plot of the PL spectra taken at room temperature for various spatial positions *L* along a single ZnO NW is shown in Figure 4b, where the vertical axis represents the starting spatial position and different colors are used to differentiate the peak intensities. Obviously, with an increase of spatial position, *L* above 2.05 ± 0.05 μm, a quenching of the NIR emission was observed, attributing to the decreasing contribution of defects [39]. The origin of this NIR emission, especially the non-concomitant NIR emission, has been controversial. Some previous reports have attributed it to the transition of carriers between radiative recombination centers related to Zn interstitials and oxygen interstitials [40]. Moreover, Nguyen et al. [40] reported, at a lower growth temperature of ZnO, more interstitial zinc concentration and higher lattice disorder along the *c*-axis, which may also create deeper Zn-interstitials energy levels. The deep levels of Zn-interstitials, therefore, are attributed as the reason for NIR emission in our ZnO nanorods. The most widely accepted origin of the NIR emission is typically associated with oxygen deficiency; orange emission is associated with excess oxygen [41,42,43]. Based on a recent calculation on the transition levels of the native point defects of ZnO [41], the NIR emission can be successfully explained by the donor-acceptor transition between oxygen- and zinc-vacancies and/or the radiative recombination of shallowly trapped electrons with deeply trapped holes at oxygen interstitials. In the present work, the broad NIR emission band could originate from surface defect related states such as the recombination of deep trap electrons with free valence band (VB) holes or holes in shallow traps, as shown in Figure 4c using Kröger-Vink notation; Zn = zinc, O = oxygen, and V = vacancy. The terms indicate the atomic sites, and superscripted terms indicate charges, where a star indicates positive charge and a prime indicates negative charge, with the charges in proportion to the numbers of symbols [44]. A dominant process of ZnO NW for the NIR emission may depend on the concentration of the native defects that are present. The proposed recombination processes in Figure 4c can successfully explain the NIR luminescence from ZnO, although there is no direct evidence of Zn-vacancies in this work. However, further investigation of the related experiment is required to interpret the existence of zinc vacancies. The recombination of an electron-hole pair can exist as a Wannier exciton and recombines a radiative photon that creates a sub-band energy close to the NIR regime in the ZnO NW. The defect states are associated with the deep-trapped electron $V_{O}^{**}$ and the shallow-trapped hole $V_{Zn}^{\prime \prime }$ of the ZnO NW and follow energetic shifts analogous to the degree of excitonic confinement. To understand the confinement effect, the correlation length of the mean distance between such defects in the sub-band-gap of ZnO NW from the phonon E_2_^H^ mode profile can be calculated from a linear chain model modified from the phonon confinement model. The interplay of correlation length between defects mediating the NIR emission and the phonon vibration mode is likely fundamental to the 1D ZnO NW system, as drawn in a schematic plot of Figure 4d.

### 2.5. Linear Chain Modeling and the Phonon Peak of E_2_^H^ Mode Mapping

In general, a phonon of wave vector can interact with photons and electrons, and the scattering process is governed by a selection rule [45]. In an infinite lattice, the selection rule (q ~ 0) is satisfied. When the size is reduced, for example, to the nanoscale, the rule would be broken [45,46,47,48]. Since the phonons have been confined to a limited region by boundaries or defects, the rapidly decreasing wave function results in a wavevector uncertainty in the center of the Brillouin zone and a corresponding frequency uncertainty [36]. Then the extra contribution of light scattering, which takes place from the quasi-zone-center, will lead the phonon curves to become asymmetric and broadened [49,50]. The changes in phonon propagation would lead to different properties than bulk. Therefore, many studies have focused on the phonon confinement effect in nanomaterials. The Raman scattering intensity for the phonon confinement effect can be derived as follows. Let us consider that the phonon is confined in a column nanowire with a mean distance of defects $\xi_{L}$. When the size is small enough, a phonon cannot propagate as a plane wave in bulk. A confinement factor W(r) must be considered in the wave function to let the frequencies decay close to the boundary value. The wave function $\psi \left(q_{o},\;r\right)$ of a confined phonon is expressed as [51]:

(1) $$\psi \left(q_{o},r\right)=W\left(r\right)\cdot u\left(q_{o},r\right)\cdot \exp\left(-iq_{o}\cdot r\right)=\psi^{\prime }\left(q_{o},r\right)\cdot u\left(q_{o},r\right),$$

where $q_{o}$ is the scattering wavevector q ~ 0, *r* is the distance, and $u\left(q_{o},r\right)$ is the factor of lattice periodicity. The wave function can be expanded in a Fourier series:

(2) $$\psi^{\prime }\left(q_{o},r\right)=\int C\left(q_{o},q\right)\cdot \exp\left(iq\cdot r\right)d^{3}q,$$

The coefficient is as follows:

(3) $$C\left(q_{o},q\right)={\left(2\pi \right)}^{-3}\int \psi^{\prime }\left(q_{o},r\right)\cdot \exp\left(-iq\cdot r\right)d^{3}r,$$

The Gaussian function is frequently used as the confinement factor: if $W\left(r\right)=\exp\left(-\frac{\alpha r^{2}}{\xi_{L}^{2}}\right)$, the coefficient can be written as $C\left(q_{o},q\right)=\exp\left(-\frac{\xi_{L}^{2}{\left(q-q_{o}\right)}^{2}}{4\alpha }\right)$, where α is related to the convergence rate of the function. Concerning the value of α, $\alpha =4\pi^{2}$ is a good candidate for the phonon confinement factor of Raman intensity in TiO_2_ nanocrystals [50], silicon nanowires [38,52], porous silicon [53], GaAs nanowires [50], ThO_2_ nano-powders [54], and ZnO nanoparticles [55]. As one-phonon Raman scattering probe zone-centers ($q_{o}=0$), the square of the coefficient can be shown as ${\left|C\left(0,q\right)\right|}^{2}=\exp\left(-\frac{q^{2}\xi_{L}^{2}}{16\pi^{2}}\right)$. The intensity of the first-order Raman intensity is expressed as:

(4) I(w)≃∫|C(0,q)|2[w−w(q)]2+(Γ2)2d3q,

where w(q) is the phonon dispersion relation and Г is the full width at the half maximum of the E_2_^H^ phonon peak of ZnO bulk (Г = 10.3 cm^−1^). The dispersion relation is usually simplified to be isotropic. According to the previous report by Begum et al. [49], the dispersion relation for diatomic molecular systems such as ZnO, based on the linear-chain model, could be written as:

(5) $$w\left(q\right)=C\sqrt{\frac{M_{1}+M_{2}+\sqrt{M_{1}+M_{2}+2M_{1}M_{2}\cos\left(qa\right)}}{M_{1}M_{2}}},$$

where *C* is the force constant between nearest-neighbor planes, a is the lattice constant, and *M*_1_ and *M*_2_ are the atomic weights of various elements. Figure S2 (see supporting information) shows a simulation of Raman curves at various correlation lengths. It can be seen that the peak shows a downshift and asymmetric broadening as the correlation length decreases. In the nanowire, the diameter is much smaller than the mean diameter of ZnO NW. It is worth noting that phonon confinement mainly occurs along the radial direction [48,56] and the contribution from the axial direction is negligible. The equation for Raman intensity can be rewritten as [57]

**Figure 5:**
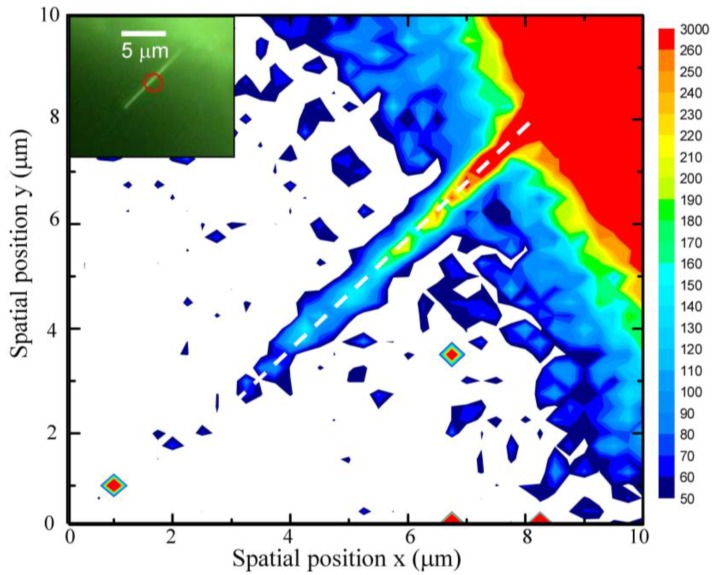
Raman intensities mapping of the E_2_^H^ peak ranging from 410 to 460 cm^−1^ and (inset) the corresponding optical image.

**Figure 6:**
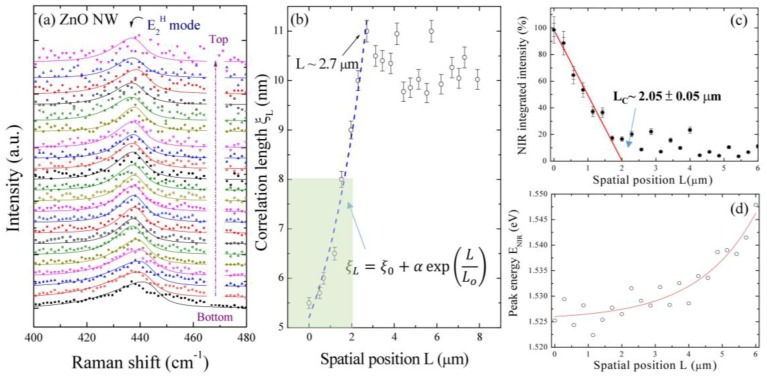
(**a**) A series of Raman spectra of the E_2_^H^ mode taken along the growth direction; (**b**) Correlation lengths versus the spatial position along the ZnO NW. Spatial position dependencies of (**c**) integrated intensity of NIR emission and (**d**) NIR sub-band gap energy. The solid red lines represent a linear fit to the intensity data and an exponential fit to the emission energy, respectively.

(6) I(w)≃∫|C(0,q)|2[w−w(q)]2+(Γ2)22πqdq,

Also, the model would be invalid as the correlation length is less than 5 nm because the influence of the Brillouin zone shape cannot be ignored [51]. A Raman mapping (10 × 10 µm^2^) of the integrated intensity of the E_2_^H^ mode of a selected single ZnO NW integrates a range from 425 to 450 cm^−1^. Here different colors are used to distinguish the integrated intensity, while the color bar on the right-hand side reveals the magnitude of the integrated area. The colored area reflects the existence of zinc oxide. A higher intensity was observed near the bottom of the ZnO NW, revealing the ZnO film on the Ti grid surface. The decrease of integrated intensities along the growth direction should be related to the reduced scattering source and tilted ZnO NWs. Moreover, the blue color at the edge of the ZnO NWs is attributed to the surface polarization effect [38]. A series of images of the E_2_^H^ mode was taken along the growth direction from bottom to top (marked with a dashed line in Figure 5). As can be seen in the bottom region of Figure 6a, an obvious asymmetric profile reflects that the phonon is mainly confined by small local domains related to the mixed state of Zn-interstitials and/or oxygen vacancies, not by boundaries. This observation is consistent with the previous analysis of short circuit diffusion for the growth mechanism along a ZnO NW [25]. Figure 6a shows a series of the E_2_^H^ mode Raman data and fitting curves along the growth direction from the bottom to top for the ZnO NW, in which the square is the experimental data, and the solid line is the fitting curve. The fitting range in wavenumbers is from 400 to 480 cm^−1^. It can be seen that the fitting curves are approximately consistent with the experimental data. Some causes result in these imperfect fittings. One is that the strain effect caused by zinc and oxygen vacancies gives a contribution on the high-frequency side. The other is the scattering peak of the E_1_-TO mode (at 411.9 cm^−1^ in bulk) near the E_2_^H^ mode (at 436.7 cm^−1^ in bulk). As the peak width becomes quite large, it is difficult to distinguish the contribution on the lower frequency side from the phonon confinement effect or the E_1_-TO mode. Figure 6b displays the obtained correlation lengths $\xi_{L}$ against the spatial positions *L* along the ZnO NW. The short range correlation lengths $\xi_{L}$ along the growth direction are estimated to be about 5.5 to 11 nm for the ZnO NW, following an exponential growth law below *L* < 2.7 µm. The solid red curve indicates the fit of the data, namely $\xi_{L}=\xi_{o}+\alpha \exp\left(\frac{L}{L_{o}}\right)$, where $\xi_{o}$ = 4.1(1) nm, α = 1.2 ± 0.1 nm, and *L_o_* = 2.7 ± 0.2 µm represent the initial constants and the fitted parameters, respectively. Compared with the mean diameter of 36 ± 4 nm, an existence of abundant defects was assumed in the ZnO NW. The result reveals that the phonon is mainly confined by defects, not by boundaries. The increasing nucleation of the correlation lengths indicates the decreasing concentration of the defects; thus the intensity of the NIR luminescence will also be influenced. As shown in Figure 6c, the normalized integrated intensity of NIR emission, with respect to spatial position, reveals a decreasing trend, with an increase in spatial position in the region of 0 < *L* < 2 µm. The solid line represents a linear function fit to the data, showing a slope of 50% ± 4%/µm. Extrapolating the linear fit to a zero intensity percentage gives a critical spatial position of *L*_C_ ~2.05 ± 0.05 µm and a correlation length of $\xi_{L}$ ~8 nm (as can be seen in green block of Figure 6b), above which, due to the decreasing number of defects, the concentration resulting in NIR emission vanished. The obtained critical short-range correlation length of $\xi_{L}$ ~8 nm reveals that the phonon confinement effect will enhance the recombination process of the deep-trapped electron $V_{O}^{**}$ and the shallow-trapped hole $V_{Zn}^{\prime \prime }$ of the ZnO NW. Additionally, the NIR luminescence peak energy displays a blue shift compared to the NIR Peak at *L* = 0, as shown in Figure 6d. The peak energy depicts the exponential dependency of the spatial position, where the solid line is the fit to the data. The fitting parameter yields the spatial position *L* = 0, having a critical band gap energy of 1.53 ± 0.01 eV. The observed enhanced NIR emission energy with the increase of the spatial position can be attributed to the double-ionized oxygen vacancies, which spontaneously transform into stable neutral and single-ionized oxygen vacancies. Since the neutral and singly-ionized oxygen vacancy occupied higher energy levels, this results in a larger NIR energy subband gap. Therefore, a small correlation length $\xi_{L}$, with high oxygen vacancies on the surface, that has a high electron population in valence band, when excited, will result in enhanced intensity due to an increased electron hole recombination process. On the other hand, in stoichiometric analyses, a larger correlation length $\xi_{L}$, reducing the intensity of NIR sub-band emissions, will be observed due to a decreased electron hole recombination process.

## 3. Discussion

ZnO NWs are of great interest due to their one-dimensional confinement. It had been reported that the exciton-photon coupling in a ZnO NW is particularly strong compared to bulk ZnO. However, since ZnO exhibits significant exciton binding energy, the Bohr radius of its excitons is very small (~1.8 nm). The quantum confinement effects would not occur until the sizes of the nanostructures were comparable with the Bohr radius. The small exciton radius of ZnO leads to difficulty in the observation of quantum confinement effects in the ZnO nanostructures. The confinement of optical phonons in nanowires (<10 nm) results in asymmetrically broadened Raman lines, which are shifted toward lower wavenumbers. The phonon confinement model relates the observed changes in the Raman spectra to the crystal size and defects and can be used for defect characterization at the nanoscale. In the analysis of short range correlation lengths $\xi_{L}$ from a linear chain model, it is revealed that the obtained mean distance of defects in the range of 5 to 8 nm (as shown in Figure 6b of the green shadow regime) are uniformly distributed in a crystallized ZnO NW with diameters of 36 ± 4 nm. The phonon confinement effect of the ZnO dots was well manifested by a significant blue shift of about 5 to 10 meV in the photoluminescence (PL) spectrum at room temperature. Native or intrinsic defects are imperfections in the crystal lattice that involve only the constituent elements, including vacancies (missing ions at regular lattice positions) and interstitials (extra atoms occupying interstices in the lattice). During the un-element-doped and catalystless ZnO NW growth process in CVD method, it is more favorable to generate a vacancy than interstitial defects, if an energy and chemical balance between the nanowire and the short circuit diffusion is considered. Therefore, Zn-interstitials and/or oxygen vacancies can be formed simultaneously and could be the most probable candidates for NIR sub-band emission. Understanding the incorporation and behavior of point defects in ZnO is therefore essential to its successful application in semiconductor devices. In this present work, EDS-, confocal Raman-, and PL-mapping techniques were used to characterize the defect concentration along the growth direction of a single ZnO NW, showing the possible related defect effects in different regions of a single ZnO NW. A mixed defective state length composed from the Zn-interstitials and/or oxygen vacancies was observed at the bottom region of ZnO NWs. At the middle and top regions of a single ZnO NW, the phonon confinement effect and the strain effect are mainly responsible for the peak shift by analyzing the E_2_^H^ mode. A theoretical approach to chain modeling modified from phonon confinement and a Raman mapping technique, are combined to give new insight into the calculation of the correlation length of the mean distance between such defects in the sub-band-gap of ZnO NW, where the correlation length gradually increased until the NIRemission vanished. As the correlation length *ξ_L_* became greater than 8 nm, a blue-shift of NIR energy was observed, and the number of electron-hole pairs is found to decrease continuously, while the mean separation between electrons and holes within the remaining bound pairs is increased. Future experimental studies that focus on the dependence of rotation and tilt angels on a single nanowire [58] to obtain more detailed information about its properties may help to identify the mechanism associated with the photonic and phononic properties in ZnO NWs.

## 4. Method

In-plane ZnO NW, without an auxiliary template or any catalyst, were prepared using a chemical vapor device method. A 150-mesh titanium grid was selected as a substrate candidate due to both zinc and titanium having hexagonal structures, with a space group of *P6_3_/mmc* and lower lattice mismatches. Compared with other methods, this approach is a simple, convenient, and reliable method for preparing in-plane ZnO NWs. The Ti-grid with Zn ingots was put on a porcelain boat in a quartz tube furnace with annealing temperatures (*T*_A_) of 500 °C for 2 h, as shown in Figure S3a. After the temperature was stabilized, a mixed gas of oxygen (20 *sccm*) and argon (80 *sccm*) was introduced into the tube, and the pressure was kept at 760 Torr by a flux controller. The zinc atoms combined with the oxygen atoms, after which in-plane ZnO NWs grew on the ZnO film, as shown in Figure S3b. Since the ZnO nanowire growth process is dependent on the surface diffusion of Zn (with respect to the annealing temperature and time), the cross-section of such a Ti-grid should look like the core-shell structure of Ti-grid → Zn film → ZnO nanowire. To verify the formation of a ZnO NW, further SEM investigation was carried out. An energy dispersive spectroscopy (EDS) (Inca X-sight model 7557 Oxford Instrument, Abingdon, Oxfordshire, UK) mapping technique was used to measure the distributions of Ti, Zn, and O. EDS mapping generates a two-dimensional image, indicating the abundance of an element. An EDS detector scans along a ZnO NW surface and then depicts a corresponding element mapping of the O, Zn, and Ti concentrations. The morphology and size distribution analysis were carried out by using field-emission scanning electron microscopy (FE-SEM, JEOL, JSM 6500F, Peabody, MA, USA) microscope. Transmission electron microscopy (TEM, JEOL, JEOL 3010, Peabody, MA, USA) is an important microscopic technique for cancer research, material science, and nanotechnology. In the present study, the high-resolution (HR) images and the selected area electron diffraction (SAED) patterns were obtained by analytical transmission electron microscopy. In these Raman and PL measurements, the phonon vibration modes were characterized using confocal Raman spectroscopy (Alpha 300, WiTec Pte. Ltd., Ulm, Germany), equipped with two scanning systems: a motorized stage for coarse movements and a piezo stage for sub-nanometer adjustments. The samples were excited with a 488-nm Ar ion laser (CVI Melles Griot, Carlsbad, CA, USA) (0.05 mW laser power) to form a spot 0.3 μm in diameter, giving a power density of 100 mW·cm^−2^. The schematic plot of the Raman spectroscopy is shown in Figure S4. The high linear, piezo-driven, and feed-back controlled scan stage can allow automated and high-resolution Raman mapping experiments and large area investigations. The confocal Raman spectroscopy has an optical resolution of 200 nm laterally and 500 nm vertically, and a spectral resolution down to 0.02 wavenumbers was used for the PL measurements. The experimental error of the spatial position has ~40 nm laterally and ~100 nm vertically, respectively. Compared with general Raman spectroscopy, the confocal setup can enhance the image contrast, reduce background interferences, and provide an in-depth investigation. The sample preparation and experimental process are described as follows. The as-grown sample was fixed on a silicon wafer with a side of ~1 cm by carbon tape. Before that, the silicon wafer as substrate was cleaned in an alcohol solution using ultrasonic cleaning for 5 min and then dried naturally in air at room temperature. The sample surface has to be flat to avoid letting the tip touch. For Raman measurements, an argon laser with a wavelength of 488 nm was used as an excitation source. After that, the laser was warmed up for at least half an hour to provide a stable power outlet, with the sample placed on a movable stage to adjust the position and height. The laser beam was introduced into a single mode fiber and then was scattered by the sample. The scattered light was reflected to a fiber and then analyzed by a spectroscopy system. Wavelengths close to the incident light due to the above-mentioned elastic Rayleigh scattering were filtered out. The confocal design can provide assurance that the scattering light from various regions will be collected to depict Raman spectra from the base along the growth direction and toward the top of a single ZnO NW.

## Figures and Tables

**Table 1:** Summary of the fitting parameters of each phonon vibration mode along the growth direction of a single ZnO NW.

Spatial Position (μm)	E_2_^H^ Mode (cm^−1^)		E_1_-LO Mode (cm^−1^)	
	Position	Width	Position	Width
0.00	438.1 ± 0.56	29.3	608.1 ± 0.54	45.4
0.50	438.2 ± 0.36	26.3	606.4 ± 0.80	44.7
0.68	437.5 ± 0.47	23.9	607.2 ± 0.62	40.4
1.20	436.8 ± 0.29	24.0	606.5 ± 1.11	51.8
1.52	436.6 ± 0.27	20.9	606.8 ± 1.27	46.7
1.97	436.6 ± 0.35	24.6	606.9 ± 1.31	44.9
2.30	436.7 ± 0.35	22.9	602.5 ± 1.76	63.1
2.71	437.3 ± 0.45	19.8	606.5 ± 2.59	55.1
3.12	436.8 ± 0.57	30.0	606.5 ± 2.36	50.6
3.45	436.8 ± 0.51	22.0	606.5 ± 2.83	44.7
3.83	436.8 ± 0.78	29.3	605.2 ± 3.54	53.7
4.13	437.5 ± 0.64	21.6	-	-
4.43	436.1 ± 0.77	21.4	-	-
4.76	436.3 ± 0.71	21.4	-	-
5.14	436.6 ± 0.59	15.8	-	-
5.53	437.2 ± 0.79	22.6	-	-
5.74	436.7 ± 0.57	13.4	-	-
6.20	434.2 ± 1.51	36.5	-	-
6.71	435.7 ± 1.04	20.8	-	-
7.03	439.6 ± 1.83	42.2	-	-
7.30	435.9 ± 1.69	24.6	-	-
7.90	439.6 ± 3.13	43.4	-	-

## Supplementary Materials

The following are available online at http://www.mdpi.com/2079-4991/7/11/353/s1, Figure S1: Confocal Raman spectrum of ZnO powders. Figure S2: Simulated Raman line shape of the E_2_^H^ mode of ZnO NW. Figure S3: A schematic plot of ZnO NW. Figure S4: A schematic diagram of confocal Raman spectrometer. Table S1: Summary of Fitting parameters of each phonon vibration mode of ZnO powder.
