# Supplementary material for: Phonon Confinement Induced Non-Concomitant Near-Infrared Emission along a Single ZnO Nanowire: Spatial Evolution Study of Phononic and Photonic Properties
Source: Nanomaterials (Basel). 2017 Oct 28;7(11):353. doi: 10.3390/nano7110353 (PMC5707570; doi:10.3390/nano7110353)
Supplement: Supplementary file 1 [file nanomaterials-07-00353-s001.pdf]

Supplementary information

Phonon Confinement Induced Non-Concomitant Near-Infrared Emission along a Single ZnO Nanowire: Spatial Evolution Study of Phononic and Photonic Properties

Po –Hsun Shih, Tai-Yue Li, Yu-Chen Yeh and Sheng Yun Wu*

Department of Physics, National Dong Hwa University, Hualien 97401, Taiwan

Figure S1 Confocal Raman spectrum of ZnO powders. The peak profiles can be described by a multi-Voigt function. The fitting results are shown in **Table S1**.

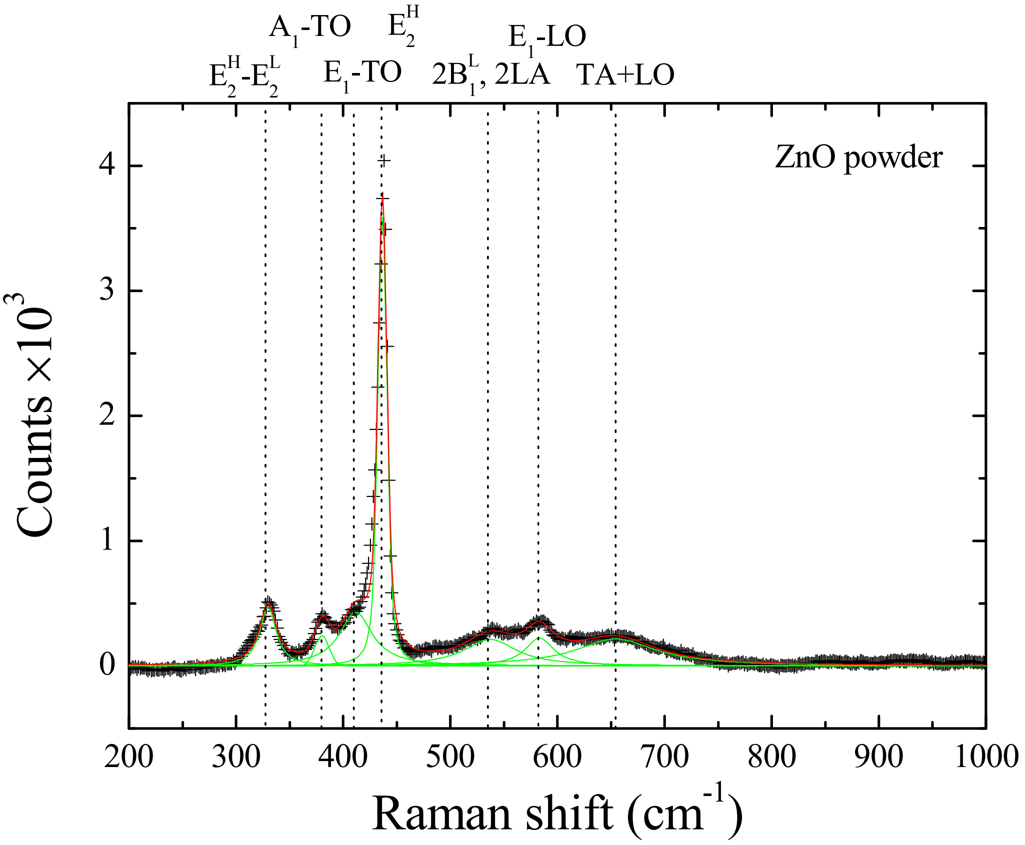

Figure S2 Simulated Raman line shape of the E_2^H mode of ZnO at various correlation length ξ_L.

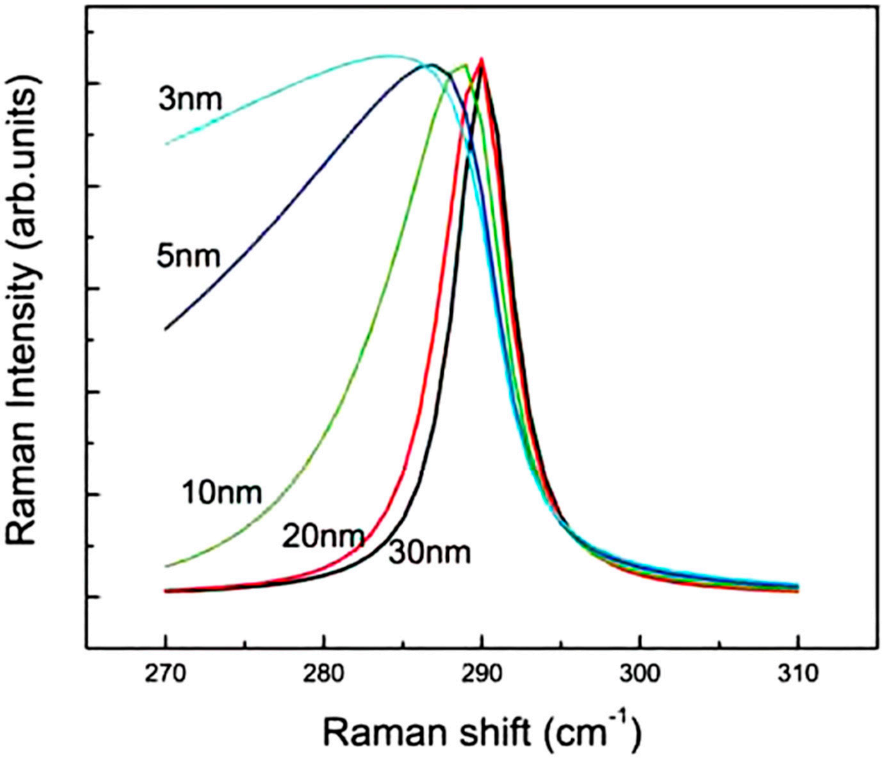

Figure S3 (a) Top view images for a porcelain boat, in which a high purity zinc ingot on a cleaned Ti-grid was mounted on a cut silicon wafer. (b) A schematic diagram of in-plane ZnO nanowires on Ti-grid.

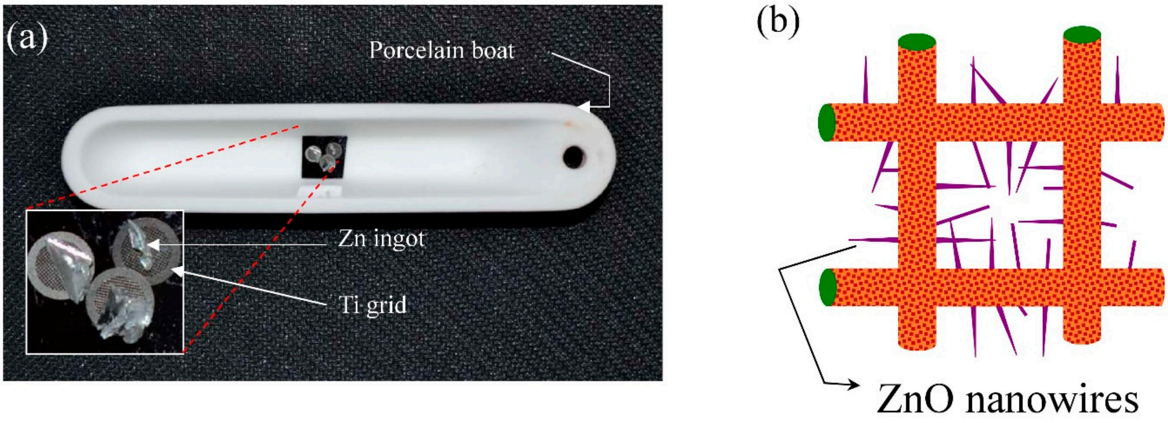

Figure S4 Schematic diagram of confocal Raman spectrometer (Wi-Tec alpha 300).

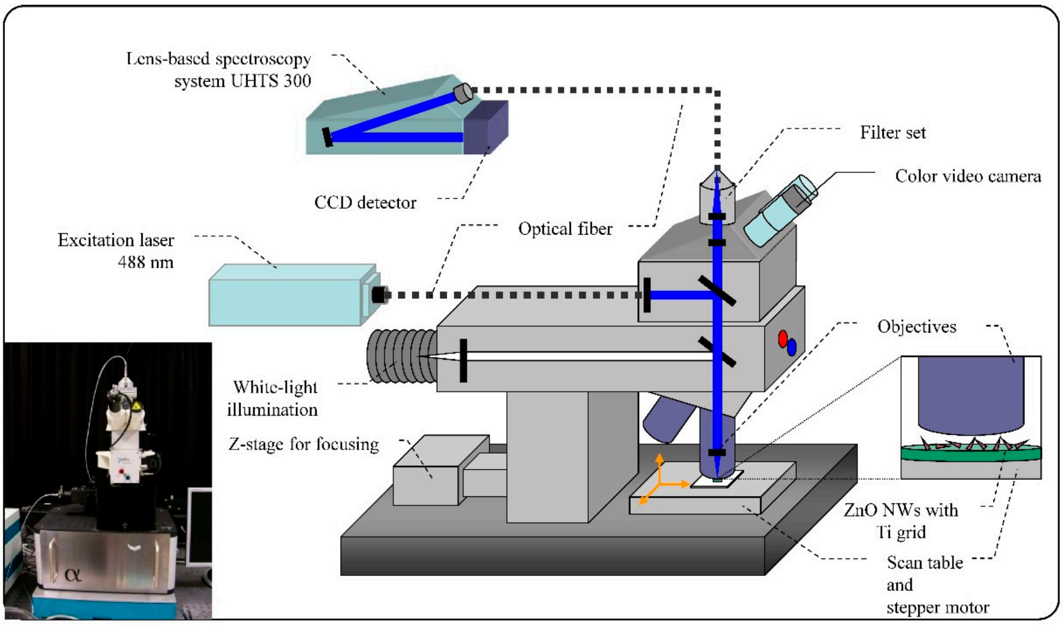

Table S1 Summary of Fitting parameters of each phonon vibration mode of ZnO powder.

Position(cm^{-1})	FWHM(cm^{-1})	Height	Area	Symmetry	Process	Ref. [23] (cm^{-1})
329.9	18.4	473	13504	A_1 (E_2, E_1)	E_2^H-E_2^L	333
380.2	15.8	242	4073	A_1	A_1-TO	378
411.9	37.5	429	25224	E_1	E_1-TO	410
436.7	10.3	3588	47703	E_2	E_2^H	438
537.2	59.9	212	19972	A_1	2LA;<br>$2B_1L$	536
583.3	32.8	222	11437	E_1	E_1-LO	590
654.5	92.0	215	31014	E_1, E_2	TA+LO	657
